# Deficits in fine motor skills in a genetic animal model of ADHD

**DOI:** 10.1186/1744-9081-6-51

**Published:** 2010-09-01

**Authors:** Yu Qian, Gefei Lei, Francisco X Castellanos, Hans Forssberg, Rochellys Diaz Heijtz

**Affiliations:** 1 Department of Neuroscience, Karolinska Institutet, S-171 77, Stockholm, Sweden; 2 Stockholm Brain Institute, Stockholm, Sweden; 3 Department of Women's and Children's Health, Astrid Lindgren Children's Hospital, Karolinska Institutet, S-171 76, Stockholm, Sweden; 4 Phyllis Green and Randolph Cowen Institute for Pediatric Neuroscience at the Child Study Center, NYU Langone Medical Center, New York, NY, USA; 5 Nathan S. Kline Institute for Psychiatric Research, Orangeburg, NY, USA

## Abstract

**Background:**

In an attempt to model some behavioral aspects of Attention Deficit/Hyperactivity Disorder (ADHD), we examined whether an existing genetic animal model of ADHD is valid for investigating not only locomotor hyperactivity, but also more complex motor coordination problems displayed by the majority of children with ADHD.

**Methods:**

We subjected young adolescent Spontaneously Hypertensive Rats (SHRs), the most commonly used genetic animal model of ADHD, to a battery of tests for motor activity, gross motor coordination, and skilled reaching. Wistar (WIS) rats were used as controls.

**Results:**

Similar to children with ADHD, young adolescent SHRs displayed locomotor hyperactivity in a familiar, but not in a novel environment. They also had lower performance scores in a complex skilled reaching task when compared to WIS rats, especially in the most sensitive measure of skilled performance (i.e., single attempt success). In contrast, their gross motor performance on a Rota-Rod test was similar to that of WIS rats.

**Conclusion:**

The results support the notion that the SHR strain is a useful animal model system to investigate potential molecular mechanisms underlying fine motor skill problems in children with ADHD.

## Background

Attention-Deficit/Hyperactivity Disorder (ADHD) is one of the most prevalent neurodevelopmental disorders, affecting approximately 3-9% of all school-aged children [1]. It is characterized by a persistent, developmentally inappropriate pattern of hyperactivity, inattention and impulsivity that impairs academic performance, social interaction, and family function. A high percentage of children with ADHD (30-50%) continue to experience symptoms through adolescence and into adulthood [2]. Besides the cardinal symptoms of ADHD, poor motor coordination or motor performance commonly co-occurs in children with ADHD. The wide range of motor problems includes excessive overflow movements, poor timing, force control and greater variability in motor outcomes, poor balancing, difficulties in both learning and performing a variety of motor skills (e.g., tying shoes, playing sports), and deficits in fine motor skills (e.g., poor handwriting) (see [3] and references therein). Clinical and epidemiological studies indicate that up to 50% of children with ADHD display motor coordination problems consistent with developmental coordination disorder (DCD) [4-9]. The typical feature of DCD involves a marked impairment in the performance of motor skills that is not due to general intellectual, sensory, or motor neurological impairment. Despite the linking of poor motor performance to poor academic performance and social functioning in children and adolescents, the co-occurrence of poor motor abilities in ADHD has received little attention in both clinical and experimental research.

Among the existing rodent models of ADHD, the Spontaneously Hypertensive Rat (SHR) is the best-characterized genetic animal model of ADHD [10,11]. The SHR strain displays the major symptoms of ADHD, including inattention, impulsivity, and hyperactivity when compared to its progenitor strain, the Wistar-Kyoto (WKY). Despite the considerable appeal of this model, several concerns have been raised [12-14]. In particular, the use of the WKY strain as control since it shows several behavioral abnormalities (e.g., hypoactivity and depression-like phenotype). However, the SHR strain has not been previously examined as a model for deficits in fine motor skills in ADHD, leaving this aspect of the model understudied. In the present study, we took advantage of a well-validated rat skilled reaching task that has been widely used to model neurological conditions such as Parkinson's disease [15], to investigate whether the SHR strain is valid for investigating not only locomotor hyperactivity, but also more complex motor coordination problems displayed by the majority of children and adolescent with ADHD. Importantly, the Wistar (WIS) strain was used as control since this strain is more active than the WKY strain.

## Methods

### Subjects

All experiments were performed in 30 to 35-day-old SHRs (SHR/NCrl) from Charles River, Sulzfeld, Germany and WIS (Wistar/Furth/Sca) from Scanbur AB, Sollentuna, Sweden. Both the SHR and WIS rats are maintained as inbred colonies. The animals arrived in the laboratory one week before the experiment (i.e., when they were 23 to 25-day-old) and were housed in groups in standard plastic cages (Type IV Makrolon^®^) under controlled conditions of light: dark cycle (12:12 h, lights on at 07.00 h). Food and water were available *ad libitum*. Animals involved in the skilled reaching task were housed in pairs in the same type of cages. The pair housed rats were separated by a clear Plexiglas partition (containing small holes; 15 mm diameter), dividing the home cage in half. All procedures were approved by the local Committee on Ethics of Animal Experimentation, Stockholm, Sweden.

### Feeding and food restriction

Prior to and during skilled reaching training, 35-day-old rats were put on a reduced diet until they reached 90-95% of their body weight. To familiarize the rats with the target food, each rat received twenty 45 mg dustless banana flavor precision pellets (Bioserv Inc., Frenchtown, NJ, USA) 8 hrs prior to the daily Purina rat chow ration the week preceding training. Once skilled reaching training began, and until the end of the study, only rat chow was served in the home cage.

### General behavioral procedure

Testing took place between 09.00 and 16.00 h under low illumination in order to reduce stress (this was critical especially for the open field test). Prior to all behavioral procedures, animals were brought in their home cages to a room adjacent to the testing room, and allowed to rest for 1 hr before testing. Care was taken to minimize stress during transportation and handling. In order to avoid carry-over effects, independent groups of animals were used in each of the different behavioral tasks (N = 5, 8, and 8 per strain for activity/open field test, skilled reaching behavior and motor coordination and balance test, respectively).

### Activity/open field test

Naïve animals (N = 5 per strain) were placed individually in the center of an activity box (48 cm × 48 cm; Acti-Mot detection system, TSE, Bad Homburg, Germany) and their spontaneous activity was measured for 60 min as previously described [16]. The following parameters were automatically recorded by the computer program: distance traveled in the center, periphery, and total (entire box), as well as the number of rearing activity (vertical photo beam breaks) and time spent in slow (>5 cm/s) or fast (>20 cm/s) locomotion.

### Skilled reaching test

#### Single pellet reaching box

Single pellet reaching boxes were made of clear Plexiglas (14 cm wide, 45 cm long and 35 cm high) as previously described [15]. In the center of each box's front wall, there was a vertical slot 1 cm wide that extended from 2 cm above the floor to a height of 16.5 cm. In front of the slot, a shelf (width 4.5 cm and length 13 cm) was mounted 3 cm above the floor on the outside of the wall. Small indentations to hold the food targets were located 2 cm from the inside of the front wall, aligned with the edges of the slot. This location prevents the rat from lapping the food with its tongue.

#### Video recording

Reaching performance was video recorded with a SAMSUNG HMX-H100P high definition camcorder. Frame-by-frame analysis was provided by computer-based software (Cyberlink Power DVD 9).

### Pre-training and training

Three days prior to skilled reaching training, animals were placed daily into the reaching cage with food pellets (see above) on the shelf for 15 min. The objective was to introduce the rat to the testing box and to have the rat retrieve the food pellet by paw or tongue. Once a rat consistently retrieved food pellets, the pellets were positioned farther away on the shelf in order to encourage paw use. By the end of the third day, approximately 90% of the animals demonstrated a consistent preference for one paw by making more reaching attempts (>80% of the time) with it. At this stage, individual food pellets were placed into the indentation contralateral to the preferred paw. During the subsequent 13 days, rats continued to receive daily 15 min training sessions, consisting of discrete trials (i.e., 10 pellets for warm up and 20 pellets for scoring). The food pellet was immediately removed from the shelf when the rat displaced it farther away from the indentation (i.e., an unsuccessful trial) to prevent additional reaching attempts. During inter-trial intervals, rats were trained to leave the slot, walk to the rear wall of the cage, and wait a few seconds before returning to the front of the cage for the next food pellet. This was accomplished by occasionally placing a food pellet close to the rear wall of the cage. In addition, food pellets were withheld from the shelf on semi-randomly selected trials in order to teach the animals to reach only if a food pellet was present in the front shelf. Thus, each rat eventually learned to orient to the food pellet, transport its limb through the slot, grasp the food pellet, retract its paw through the slot to release the food into its mouth, and then, leave the slot, walk to the rear wall of the cage, and wait a few seconds until the next trial.

### Endpoint analysis of reaching behaviour

Reaching behaviour was analyzed by measuring:

(1) Total success. A successful reach was defined as one in which an animal grasped a food pellet, transported it in the paw into the cage, and placed it into its mouth regardless of the number of limb advances toward the food pellet required. Total success was calculated as: Success % = (number of pellets obtained/20) × 100.

(2) First attempt success. First attempt success was the percentage of success in which a rat obtained a food pellet on the first advance of the limb toward the food First attempt success was calculated as: Success on 1^st ^reach % = (number of pellets obtained on first advance/20) × 100.

(3) Total number of attempts. Total number of attempts included all movements of the paw toward the food pellet (e.g., limb movements towards the slot, movements touching the shelf, and movements of the paw through the slot).

### Motor coordination and balance test

Motor coordination and balance were tested using an accelerating Rota-Rod (UGO Basile Accelerating Rota-Rod). One day before testing, rats were accustomed to the Rota-Rod by being placed on the drum rotating at low speed (i.e., four to five R.P.M.) for two 90-second periods, two hours apart. The Rota-Rod test was performed by placing a rat on the rotating drum and measuring the time each animal was able to maintain its balance walking on top of the rod. The speed of the Rota-Rod accelerated from four to forty R.P.M. over five minutes.

### Statistical analysis

All behavioral experiments were analyzed using either repeated measures analysis of variance (ANOVA; Strains and Time as main factors) or factorial ANOVA. When ANOVA indicated a significant overall effect of treatment at P < 0.05 level, post hoc testing was performed using Fisher's least significant difference (FLSD) test. All data are presented as mean ± S.E.M.

## Results

### Activity/open field test

**Figure 1:**
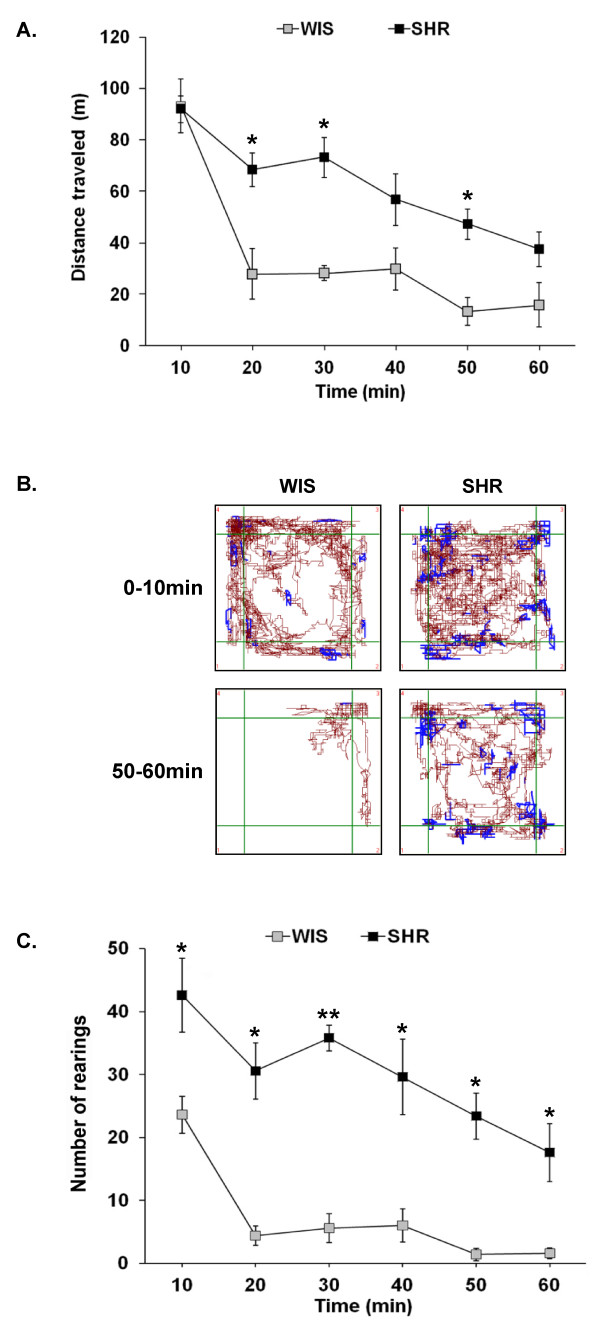
**SHRs show increased locomotor and rearing activity**. Naïve SHR and WIS rats were exposed to an activity/open field box and their spontaneous motor activity was recorded for 1 h. (A) Distance traveled (meters) as a function of time during the 60 min open field test. (B) Representative tracks of movement patterns of SHR and WIS rats during the initial 10 min of testing and at the 50-60 min time interval of testing. Distance traveled and rearing activity are shown in brown and blue colors, respectively. (C) Number of rears as a function of time during the 60 min open field test. All data (A and C) are represented as mean ± S.M.E. (N = 5 animals per group). *P < 0.05, **P < 0.001 when compared to WIS rats.

Naïve rats were placed into an activity/open field testing box and their locomotor and rearing activities were measured for 60 min. As shown in Figure 1A, SHRs travelled significantly (F (1, 8) = 18.93, P < 0.01) farther than WIS rats. Further analysis revealed that both strains of rats displayed similar locomotor activity during the initial period of testing (i.e., first 10 min; Figure 1A). In addition, locomotor activity did not differ in the different areas of the open field box: center (WIS: 36 ± 8, SHR: 48 ± 8; P > 0.1) and periphery (WIS: 60 ± 5, SHR: 44 ± 8; P > 0.1). However, significant strain differences were detected in habituation over time (strain by time interaction; (F (5,40) = 2.985, P < 0.05). Thus, SHRs traveled a significantly longer distance (Figure 1A) and spent significantly (P < 0.05) more time (minutes) in both slow (WIS: 5.14 ± 0.71, SHR: 13.43 ± 1.16) and fast locomotion (WIS: 2.08 ± 0.27, SHR: 5.88 ± 0.52), during the habituation period than WIS rats. Throughout the testing period, SHRs also reared significantly (F(1,8) = 42.404, P < 0.001) more than WIS rats (Figure 1C).

**Figure 2:**
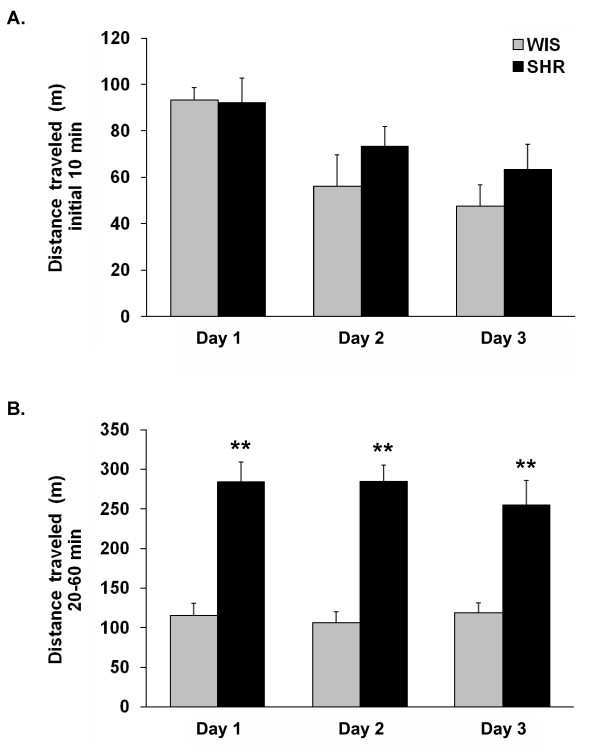
**SHRs are hyperactive and have decreased response habituation**. Naïve SHR and WIS rats were exposed to an activity/open field box for 1 h on day one (see Figure 1) and were re-exposed to the same testing box for two consecutive days. (A) Average distance traveled (meters) during the initial 10 min of testing. (B) Average distance traveled (meters) during the 20-60 min interval of testing. All data are represented as mean ± S.M.E. (N = 5 animals per group). *P < 0.05, **P < 0.001 when compared to WIS rats.

**Figure 3:**
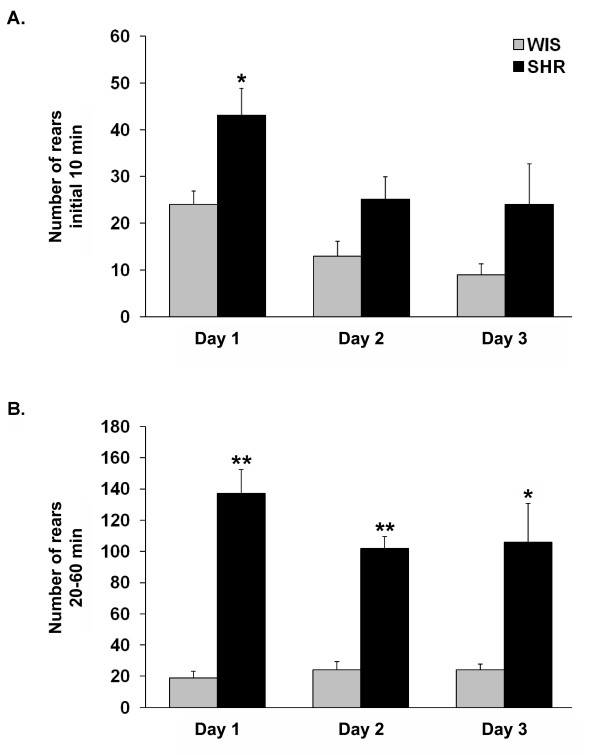
**SHRs show increased levels of rearing activity**. Naïve SHR and WIS rats were exposed to an activity/open field box for 1 h on day one (see Figure 1) and were re-exposed to the same testing box for two consecutive days. (A) Average number of rears during the initial 10 min of testing. (B) Average number of rears during the 20-60 min interval of testing. All data are represented as mean ± S.M.E. (N = 5 animals per group). *P < 0.05, **P < 0.001 when compared to WIS rats.

After their initial exposure to the activity/open field box, rats were re-exposed to the same box for 2 additional consecutive days (Figures 2 and 3). Similar to the first day of testing, the two strains did not differ significantly in distance traveled during the initial testing period (i.e., first 10 min of testing; Figure 2A) on day two or day three of testing, although both SHR and WIS rats showed a significant (P < 0.05) reduction in activity on the second day of testing. In terms of the rearing activity, there was a tendency for SHRs to rear more than WIS rats on day two of testing (P = 0.076; Figure 3A). However, no significant differences were found on day three of testing. During the habituation period (i.e., 20-60 min of testing), significant strain differences were observed on day two and day three of testing, with SHRs displaying both greater distance traveled (Figure 2B) and number of rears (Figure 3B) compared to WIS rats.

### Endpoint measures of skilled reaching

**Figure 4:**
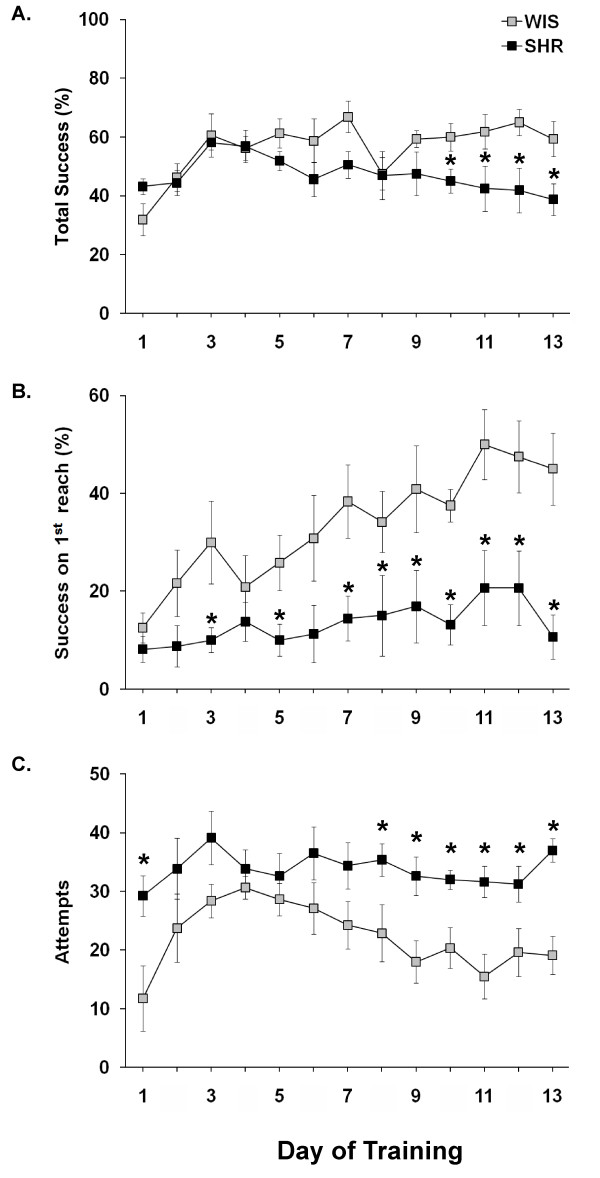
**Deficits in skilled reaching behavior in SHRs**. Endpoint measures of reaching behavior are presented: (A) Total success in percentage, (B) Success on the first reach in percentage, and (C) Attempts. *P < 0.05 when compared to WIS rats. The results are presented as mean ± S.E.M. (N = 8 per group).

Skilled reaching performance of SHRs and WIS rats is illustrated in Figure 4. The strains did not differ significantly in total success (%). However, there were significant effects of reaching training days (F (12,168) = 3.127, P < 0.001) and a significant strain by reach-training days interaction (F (12,168) = 2.205, P < 0.05). *Post-hoc *analysis showed a significant (P < 0.05) reduction in total success scores in SHRs during the last four days of training (Figure 4A).

Repeated ANOVA for success on the first reach attempt (%) revealed significant effects of strain (F(1,12) = 9.946, P < 0.01), days (F(12,144) = 5.615, P < 0.001), and a strain by reach-training days interaction (F(12,144) = 1.907, P < 0.05), with SHRs displaying very little improvement over the 13-day testing period (Figure 4B). *Post-hoc *analysis showed significantly (P < 0.05) lower scores in success on the first reach attempt (%) in SHRs on days 3, 5, and 7-13 compared to WIS rats (Figure 4B).

Evaluation of the number of attempts revealed significant effects of strain (F (1,12) = 9.946, P < 0.01), reach-training days (F(12,144) = 5.615, P < 0.001), and a strain by reach-training days interaction (F(12,144) = 1.907, P < 0.05). *Post hoc *analysis showed significantly higher number of attempts in SHRs on reach-training days 1 and 8-13 (Figure 4C).

### Motor coordination and balance test

**Figure 5:**
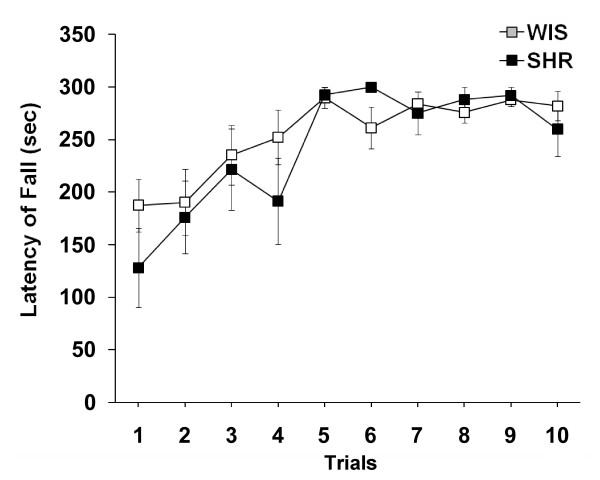
**Similar performance of young adolescent SHR and WIS rats in the Rota-Rod test**. Mean (± S.M.E.) latency to fall on indicated trials for SHR and WIS rats (N = 8 animals per group).

Performance on the Rota-Rod was measured by latencies to falling off the rotating cylinder, during 10 trials over 1 day, with a 5-min inter-trial interval. Both SHR and WIS rats improved significantly from trial 1 to trial 9 (F(9,126) = 9.121, P < 0.0001), demonstrating that both strains significantly improved motor coordination over time (Figure 5). However, no significant effect of strain (F(1,14) = 0.692, P > 0.1), nor a strain by trials interaction (F(9,126) = 0.918, P > 0.1) was found.

## Discussion

The present study provides evidence supporting the notion that the SHR strain is a useful animal model system of ADHD that allows the investigation of not only locomotor hyperactivity, but also more complex motor coordination problems displayed by the majority of children with ADHD (i.e., deficits in fine motor skills). Thus, similar to children with ADHD, adolescent SHRs had lower performance scores in a skilled reaching task when compared to control rats.

The present findings confirm and extend previous observations comparing the open field behaviour of "young adolescent SHRs to the more hypoactive WKY rats" [17,18]. In the present study, we compared the exploratory activity and habituation profile of SHRs to that of a more active strain (i.e., WIS rats) after repeated exposure to a novel environment. Rats exposed to a novel environment typically display high levels of exploratory behaviour. However, when rats are repeatedly placed into the same open field, as well as after a prolonged exposure to an open field within a session, a progressive reduction occurs in exploratory behaviour as the novel environment becomes familiar. The results of the present study showed that both SHRs and WIS rats displayed similar locomotor activity during the initial exploratory phase (first 10 min) of open field exposure, indicating that the increased locomotor activity in SHRs was not triggered by novelty. Instead, the hyperactivity of SHRs was found during the habituation phase. Similar to children with ADHD (see [10] and references therein), SHRs display locomotor hyperactivity in a familiar, but not in a novel environment.

In the current study, SHRs showed increased levels of rearing activity during both the initial novelty phase and the habituation period of open field testing. Some authors have specifically implicated rearing activity in attentive processes underlying gathering of contextual information in novel situations [19]. Thus, suggesting that non-selective attention in young adolescent SHRs is compromised.

One of the main goals of the present study was to determine whether SHRs also show the complex motor coordination problems (e.g., fine and gross motor skills) typically displayed by the majority of children with ADHD (see Background section). For this purpose, we investigated potential skill motor deficits of SHRs and WIS rats using a well-validated rodent skilled reaching task [15]. Different measures evaluated in this task provide progressively increasing sensitivity in terms of measuring skilled performance, i.e., success on first trial more than overall success (see Additional files 1, 2, 3). The results of the present study demonstrated that although SHRs can learn to perform this task, their performance is significantly poorer than that of control rats, especially in the most sensitive measure of skilled performance (i.e., success on the first trial). In fact, SHRs make multiple attempts before they can grasp and eat a pellet (see Additional file 4). The lower performance of SHRs does not appear to be due to the abnormality or absence of a reach sequence (advance-grasp-withdrawal-release). Although we could not quantify the speed of forelimb movements in our animals, we noticed that SHRs make faster forelimb movements than control rats. Interestingly, studies have demonstrated that children with ADHD perform jerky arm movements and show a reduced capacity to select a movement speed appropriate to the accuracy demands of the task [7].

It is worth mentioning that although hand shaping movements of release, collection, and manipulation in skilled reaching are similar in humans, monkeys, and rodents [20,21], rodents display high individual variability in skilled movement success in reaching for food [22]. In the present study, we also observed such individual differences in skilled reaching behaviour in both strains. In agreement with previous studies [23], gross motor coordination (as evaluated on the Rota-Rod) appears to be normal in SHRs, suggesting that the SHR strain displays specific deficits in fine motor skills.

Several brain structures, including motor cortical regions of the frontal cortex, basal ganglia (e.g., striatum; the main input nucleus of the basal ganglia), and cerebellum are believed to be critical for the acquisition and/or consolidation of skilled motor behaviors [24-26]. These are the same regions that have been implicated in ADHD [27,28]. The neurobiology of ADHD still remains unclear, but accumulating evidence indicates a dysregulation of the dopaminergic and noradrenergic modulatory systems controlling the frontal-striatal circuits [29]. In particular, the neurotransmitter dopamine is known to play a critical role in motor performance, motor skill learning and corticostriatal synaptic plasticity [30,31]. The mechanisms underlying motor dysfunctions in young adolescent SHRs are still largely unknown. Some characteristics may be explained by alterations in pre- and/or post-synaptic dopaminergic mechanisms observed in the frontal-striatal circuit of SHRs (see [32] and references therein). Recent molecular studies have found alterations in synaptic plasticity related genes (e.g., calmodulin and calcyon) and transcription factors involved in cortical neurogenesis (e.g., Hes6) and Purkinje cell generation and differentiation (e.g., Lhx1) [33]. We have previously reported that calcyon (a risk gene for ADHD) mRNA expression is up-regulated in various frontal cortical regions (including motor cortex) and striatum of SHRs [34]. More recent evidence indicates a role for calcyon in clathrin-mediated endocytosis, a critical component of synaptic plasticity [35]. This is consistent with anatomical findings localizing calcyon to vesicular compartments in dendritic spines and axon terminals [36], two sites in the brain where clathrin-mediated endocytosis is essential for efficient neurotransmission and plasticity associated with learning and memory [35]. Further investigations using genetically engineered mice and/or *in vivo *small interfering RNA (siRNA) delivery systems might provide additional mechanistic insights regarding the potential role for calcyon [37] and other candidate genes in the acquisition and performance of fine motor skills.

## Conclusion

In conclusion, our behavioral analysis revealed deficits in fine motor skills (but not gross motor skills) in a genetic animal model of ADHD combined subtype (the SHR/NCrl rat; [11]). Hence, the results support the notion that the SHR strain is a useful animal model system to investigate potential molecular mechanisms underlying fine motor skill problems in children with ADHD. In future studies, we plan to assess whether drugs used for the treatment of ADHD (e.g., methylphenidate and atomoxetine) could ameliorate deficits in fine motor skills of SHRs and if so, identify the primary locus of their beneficiary effects.

## Abbreviations

ADHD: attention-deficit/hyperactivity disorder; SHRs: Spontaneously Hypertensive Rats; WIS: Wistar; ANOVA: analysis of variance; siRNA: small interfering RNA.

## Competing interests

The authors declare that they have no competing interests.

## Authors' contributions

YQ carried out the study and conducted the statistical analyses. YQ, GL, FXC and HF participated in the overall study design and helped draft the manuscript. RDH supervised the study and drafted the manuscript. All authors read and approved the final manuscript.

## Supplementary Material

Additional file 1 **First attempt success**. This video shows a WIS rat performing the skilled reaching task and obtaining the food pellet on the first advance of the limb (i.e., attempt) toward the pellet. Click here for file

Additional file 2 **Success on the second attempt**. This video shows a WIS rat performing the skilled reaching task and obtaining the food pellet on the second attempt. Click here for file

Additional file 3 **Failure**. This video shows a WIS rat performing the skilled reaching task and failing to obtain the food pellet. Click here for file

Additional file 4 **Success on the fourth attempt**. This video shows a SHR performing the skilled reaching task and obtaining the food pellet on the fourth attempt. Click here for file
